# Abnormal Fetal Lung of Hoxa1^−/−^ Piglets Is Rescued by Maternal Feeding with All-Trans Retinoic Acid

**DOI:** 10.3390/ani13182850

**Published:** 2023-09-07

**Authors:** Yixin Chen, Haimei Zhou, Huadong Wu, Wei Lu, Yuyong He

**Affiliations:** 1 Jiangxi Province Key Laboratory of Animal Nutrition, Engineering Research Center of Feed Development, Jiangxi Agricultural University, Nanchang 330045, China; dreazzle@163.com (Y.C.); lw20030508@163.com (W.L.); 2 Department of Animal Science, Ganzhou Polytechnic, Ganzhou 341000, China; 3 Department of Animal Science, Jiangxi Agricultural Engineering College, Zhangshu 331200, China; 18270826482@163.com; 4 College of Animal Science and Technology, Jiangxi Agricultural University, Nanchang 330045, China; whd0618@163.com

**Keywords:** Hoxa1 mutation, alveoli, pulmonary blood vessel, maternal administration, ATRA, fetal piglets

## Abstract

**Simple Summary:**

Proper development of the fetal lung is vital to the survival and healthy growth of pigs after birth, but many factors can disturb the normal growth of the fetal pig lung. In the previous study, we found that the Hoxa1 mutation of g.50111251 G > TC resulted in the congestion and edema of fetal lungs, and all neonatal Hoxa1^−/−^ piglets died of respiratory failure during the suckling period. The results of this study showed that supplementing all-trans retinoic acid (ATRA) to pregnant sows alleviated the dyspnea of neonatal Hoxa1^−/−^ piglets by increasing the IFN-γ concentration (*p* < 0.05), airspace area (*p* < 0.01) and pulmonary microvessel density (*p* < 0.01); increasing the expression of VEGFD (*p* < 0.01), PDGFD (*p* < 0.01), KDR (*p* < 0.01), ID1 (*p* < 0.01), and NEDD4 (*p* < 0.01); and decreasing the septal wall thickness (*p* < 0.01) and the expression of SFTPC (*p* < 0.01) and FOXO3 (*p* < 0.01).

**Abstract:**

Neonatal Hoxa1^−/−^ piglets were characterized by dyspnea owing to the Hoxa1 mutation, and maternal administration with ATRA alleviated the dyspnea of neonatal Hoxa1^−/−^ piglets. The purpose of this experiment was to explore how maternal ATRA administration rescued the abnormal fetal lungs of Hoxa1^−/−^ piglets. Samples of the lungs were collected from neonatal Hoxa1^−/−^ and non-Hoxa1^−/−^ piglets delivered by sows in the control group, and from neonatal Hoxa1^−/−^ piglets born by sows administered with ATRA at 4 mg/kg body weight on dpc 12, 13, or 14, respectively. These were used for the analysis of ELISA, histological morphology, immunofluorescence staining, immunohistochemistry staining, and quantitative real-time PCR. The results indicate that the Hoxa1 mutation had adverse impacts on the development of the alveoli and pulmonary microvessels of Hoxa1^−/−^ piglets. Maternal administration with ATRA at 4 mg/kg body weight on dpc 14 rescued the abnormal lung development of Hoxa1^−/−^ piglets by increasing the IFN-γ concentration (*p* < 0.05), airspace area (*p* < 0.01) and pulmonary microvessel density (*p* < 0.01); increasing the expression of VEGFD (*p* < 0.01), PDGFD (*p* < 0.01), KDR (*p* < 0.01), ID1 (*p* < 0.01), and NEDD4 (*p* < 0.01); and decreasing the septal wall thickness (*p* < 0.01) and the expression of SFTPC (*p* < 0.01) and FOXO3 (*p* < 0.01). Maternal administration with ATRA plays a vital role in rescuing the abnormal development of lung of Hoxa1^−/−^ fetal piglets.

## 1. Introduction

The lung is composed of airways, blood vessels, nerves, connective tissues, and alveoli. Blood vessels and alveoli have crucial functions in oxygen inhalation and carbon dioxide exhalation [1]. An alveolus is a tiny sac separated by a septum, and the surfaces of alveoli are lined by cells of squamous alveolar type 1 (AT1) and cuboidal alveolar type 2 (AT2) [2]. AT1 cells are responsible for gas exchanges with the surface markers of T1α and aquaporin 5 (AQP5) [3]. AT2 cells are small cuboidal cells with surface markers of surfactant protein C (SFTPC), and have metabolic, secretory, progenitor, and immunological functions [4,5]. SFTPC is a mixture of proteins and lipids that can prevent alveoli from collapsing during gas exchange.

Lung development is controlled by many factors, such as genes, nutrients, and microbiota. The results from previous experiments have shown that a decrease in the expression of pulmonary vascular endothelial growth factor (VEGF) can lead to an increase in the apoptosis of alveolar and bronchial cells [1], and maternal feeding with a vitamin A-deficient diet causes fetuses to develop lung hypoplasia [6]. Intrauterine growth retardation (IUGR) reduces the number of alveoli and the density of blood vessels in animals, increases the thickness of alveolar septa, and leads to an increase in alveolar size and a decrease in the alveolar number in animals [7]. The lack of nutrients in the alveolar stage of sheep lung development mainly damages the expression of surfactant proteins in the lungs before birth, restricts pulmonary vascular growth, reduces the alveolar surface area after birth, and thickens the gas–blood barrier [8]. In the cystic stage of lung development in intrauterine stunted mice, the possibility of gas exchange in the lungs decreased, owing to the thickened septa of the distal alveolus and the reduced elastin expression and alveolar maturation [9]. Retinoic acid (RA) is one of the bioactive metabolites of vitamin A and has vital functions in lung development [10,11]. RA can increase the number of alveolar cells in newborn animals [12], promote the activity of AT2 cells, inhibit their apoptosis, and convert AT2 cells to AT1 cells [13]. RA can also attenuate lung injury induced by hyperoxia [14], preserve the normal formation of alveoli during lung development under the condition of inadequate energy intake [15], affect the development of AT2 cells [6], and induce the formation of primordial lungs by controlling the expression of fibroblast growth factor 10 (Fgf10) [16]. The pathway of AR may play an essential role in regulating intramembranous transport across the ovine amnion into the fetal vasculature by effecting VEGF expression [17]. Early prenatal RA administration increases lung growth, restores lung maturation, and improves arterial reactivity [18], while maternal administration of RA reverses lung malformation, including its effects on the radial alveolar count, type II/type I ratio, and surfactant protein expression [19]. All-trans retinoic acid (ATRA) directly regulates the expression of AQP3 and amniotic fluid volume through binding retinoic acid receptor alpha (RARA) and death receptor 5-retinoic acid receptor element (DR5-RARE) [20].

**Figure 1:**
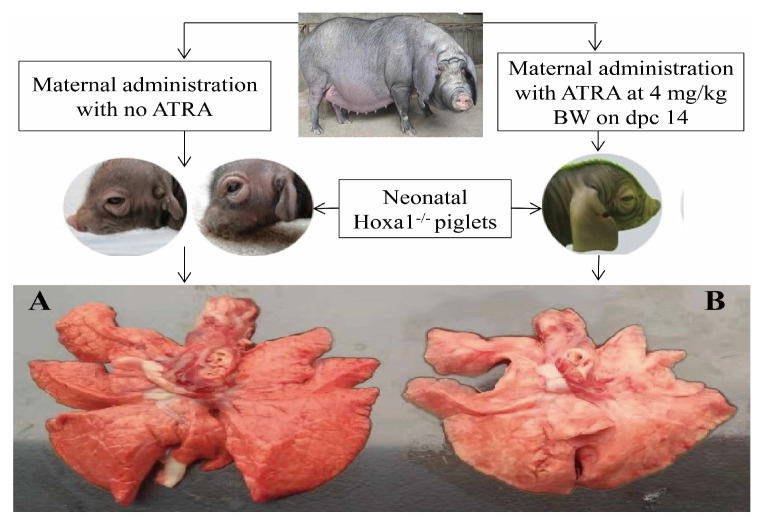
Effects of Hoxa1 mutation and maternal ATRA administration on the outward appearance of the ears and lungs of Hoxa1^−/−^ piglets.

Gene knockout or mutation damage the normal development of the lungs, and this might be related to the changed expression of genes involved in the development of blood vessels and alveoli. Knocking out an enhancer of Homeo box A1 (Hoxa1) can lead to a decrease in the expression of Hoxa1, cellular retinoic acid binding protein 1 (CRABP1), and other genes that can control the development of endoderm [21]. The Hoxa1 mutation led to a decrease in the expression of CRABP1 in fetal pigs and an increase in the expression of Cytochrome P450 26A1(CYP26A1), which decomposes retinoic acid [22], indicating that Hoxa1 may not only affect the expression of retinoic acid synthesis genes, but also affect the expression of retinoic acid transport and metabolism-related genes. In addition, Hoxa1 knockout led to the short breath and death of animals in the perinatal period, owing to the abnormal expression of genes related to early embryo endoderm differentiation [23]. Previous experiments have found that the Hoxa1 mutation caused ear deformities, dyspnea, and death of Hoxa1^−/−^ piglets shortly after birth, and the congestion and swelling of the lungs appeared after dissection (Figure 1A). In the experiment involving rescuing the ear malformations of neonatal Hoxa1^−/−^ piglets by maternal administration with ATRA at different days post coitum (dpc), we found that the abnormal respiratory symptoms and the congestion and swelling in the lungs of neonatal Hoxa1^−/−^ piglets were also alleviated (Figure 1B). The purpose of this experiment was to explore how maternal ATRA administration rescues the abnormal fetal lungs of Hoxa1^−/−^ piglets.

## 2. Materials and Methods

### 2.1. Animals and Sample Collection

Twenty-four Hoxa1^+/−^ crossbred sows (Erhualian × Shaziling) with similar body conditions were artificially inseminated with semen collected from one Hoxa1^+/−^ boar. They were randomly allocated to one control group and nine experimental groups, respectively. After mating, sows were administered with ATRA according to the treatment in Table 1: Briefly, in the morning feeding, ATRA was dissolved in dimethyl sulfoxide and diluted with soybean oil, then mixed with the regular diet at about 1/2 of the morning allowance and finally offered to sows [24].

After delivery, ear samples of all newborn piglets were immediately collected for genotyping. A total of 146 piglets (109 Hoxa1^+/−^ and 37 Hoxa1^−/−^ piglets) were euthanized with pentobarbital sodium (100 mg/kg body weight) according to the protocol approved by the Animal Ethics Committee of Jiangxi Agricultural University, and their lungs were removed. Samples of the lungs were collected from the right superior lobe, samples for morphology analysis were fixed in 4% paraformaldehyde solution, and samples for other determinations were stored at −80 °C, respectively.

### 2.2. Selection of Lung Samples

Neonatal Hoxa1^−/−^ piglets from experimental groups 1, 4, and 7 scored higher in ear development [24] and showed less hyperemia and edema in their lungs than those from other experimental groups, respectively. In addition, no differences were found in lung appearance between neonatal Hoxa1^+/−^ piglets from control group and those from the experimental groups. Based on the above findings, lung samples collected from Hoxa1^−/−^ and non-Hoxa1^−/−^ piglets in the control group and from Hoxa1^−/−^ piglets in experimental groups 1, 4, and 7 group were used for analysis.

### 2.3. Enzyme-Linked Immunosorbent Assay (ELISA)

ELISA kits produced by Elabscience Biotechnology Co. Ltd. (Wuhang, China) were used to test the concentrations of interleukin-8 (IL-8), interferon-γ (IFN-γ), and tumor necrosis factor-α (TNF-α) in the lung samples according to established protocols.

### 2.4. Histological Morphology

First, 4% paraformaldehyde solution was used to fix the lung samples for 24 h. All samples were embedded with paraffin wax, sliced into 5 μm sections, and then stained with hematoxylin and eosin (HE). Five portions were randomly selected and captured at 200 × magnification, and the airspace area and septal wall thickness were measured according to a previously reported method [25] using an Image-Pro Plus 6.0 system (Media Cybernetics, Inc., Bethesda, MD, USA).

### 2.5. Immunofluorescence Staining

Paraffin sections were firstly dewaxed and antigen-repaired, then blocked with bovine serum albumin, and finally incubated with antibody AQP5 (ABclonal, A9927) or SFTPC (GeneTex, GTX54694) in a 4 °C refrigerator overnight. After incubation, all slides were washed with phosphate-buffered saline (PBS, pH 7.4), then incubated with the secondary antibodies, washed with PBS, and stained with 4′,6-diamidino-2-phenylindole (DAPI; Servicebio, G1012) before being mounted with coverslips.

### 2.6. Immunohistochemistry Staining

Slides mounted with the 5 μm slices were treated with sodium citrate (pH 6.0) to expose antigens, followed by hydrogen peroxide (3%), and washed in PBS (pH 7.4). Slides were placed into 3% bovine serum albumin (BSA) for 30 min to block endogenous peroxidase activity and nonspecific binding of antibodies; incubated overnight with von Willebrand factor (vWF; 1:200, Dako, Carpinteria, CA, USA) at 4 °C and then incubated for 50 min with secondary antibodies; then washed in PBS (pH 7.4), finally stained with 3,3-diaminobenzidine tetrahydrochloride, hydrated, and counterstained with Harris hematoxylin.

### 2.7. Image Acquisition

A fluorescence microscope (Nikon Eclipse C1,NikonGmbH, Vienna, Austria) and a Nikon DS-U3 camera (Nikon Corporation, Shinagawa, Tokyo, Japan) were used to obtain the fluorescent images. A light microscope was applied to acquire images of the samples without immunofluorescence, and representative photomicrographs were taken for analysis using Leica QWin (Wetzlar, Germany).

### 2.8. Quantitative Real-Time PCR

The lung tissue was mechanically ground and homogenized in a mortar, and the TransZol Up Plus RNA Kit (TransGen Biotech, Beijing, China) was used to extract the total RNA. A NanoDrop 2000 spectrophotometer (Thermo Scientific, Wilmington, DE, USA) was used to evaluate the quantity and integrity of the extracted RNA.

The qualified RNA was transcribed into complementary DNA using 5 × All-In-One RT MasterMix kit (Abm, Nanjing, China). Primers were designed using NCBI’s Primer-BLAST and are summarized in Table 2. ACTB was used as the reference gene, and relative RNA expression was calculated using the 2^−∆∆CT^ method [26]. The RT-qPCR experiments were carried out in triplicate.

### 2.9. Statistical Analysis

The data were analyzed using 17.0 SPSS software (SPSS Inc., Chicago, IL, USA), and Duncan’s test was carried out to verify the statistical significance; the values were considered statistically significant at *p* < 0.05, and the results are presented as the mean ± SE.

## 3. Results

### 3.1. Concentration of Inflammatory Factors in Lung Tissue

The Hoxa1 mutation decreased the concentration of inflammatory factors in lung tissue (Table 3), and neonatal Hoxa1^−/−^ piglets had lower IFN-γ (*p* < 0.05) in their lung tissues compared to neonatal non-Hoxa1^−/−^ piglets from the control group. Supplementation of ATRA to pregnant sows at 4 mg/kg body weight on dpc 12, 13, or 14 increased the level of inflammatory factors in the lung tissue of neonatal Hoxa1^−/−^ piglets, respectively. Neonatal Hoxa1^−/−^ piglets born to sows supplemented with ATRA at 4 mg/kg body weight on dpc 14 had higher IFN-γ (*p* < 0.05) values than neonatal Hoxa1^−/−^ piglets in the control group.

### 3.2. Histological Appearances

**Figure 2:**
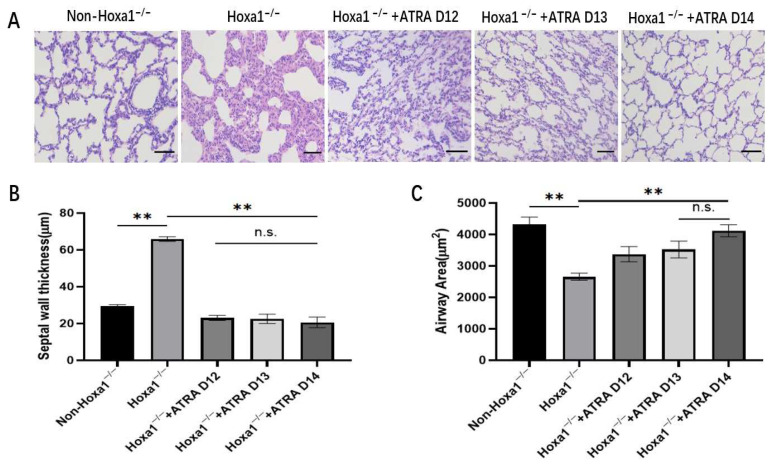
Results of HE staining. (**A**) Representative photomicrographs (×200). (**B**) Average thickness of the septal wall. (**C**) Average area of airspace. All data are expressed as mean ± SEM. ** *p* < 0.01; n.s., not significant.

The Hoxa1 mutation and maternal ATRA administration affected the lung histology of Hoax1^−/−^ fetal piglets (Figure 2). In the control group, the lungs of neonatal Hoxa1^−/−^ piglets exhibited thicker septal walls (*p* < 0.01) and smaller airspace areas (*p* < 0.01) than neonatal non-Honxa1^−/−^ piglets. Maternal administration with ATRA on dpc 12, 13, or 14 decreased (*p* < 0.01) the average thickness of the septal wall and increased (*p* < 0.01) the average area of airspaces in the lungs of neonatal Hoxa1^−/−^ piglets when compared with neonatal Hoxa1^−/−^ piglets in the control group, respectively. The lungs of neonatal Hoxa1^−/−^ piglets delivered by sows administered ATRA at dpc 14 had thinner septal walls and larger airspace areas compared with neonatal Hoxa1^−/−^ piglets born to sows supplemented with ATRA at dpc 12 or 13, respectively.

### 3.3. Development of Alveolar Epithelial Cells

**Figure 3:**
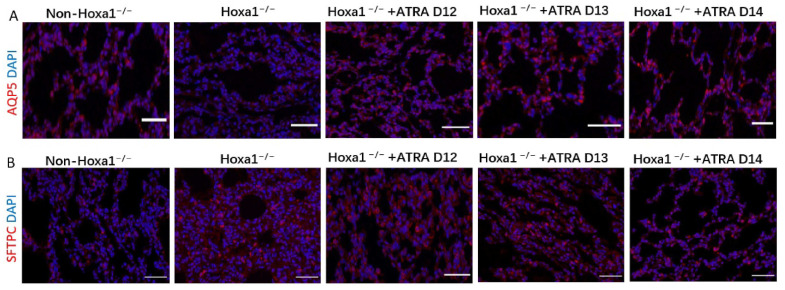
Results of immunofluorescence staining. (**A**): results of AQP5 staining; (**B**): results of SFTPC staining; Magnification: 200×. Scale bar: 30 μm.

The Hoxa1 mutation decreased AQP5 expression and increased SFTPC expression in the lungs. Neonatal Hoxa1^−/−^ piglets had lower AQP5 expression and higher SFTPC expression than neonatal non-Hoxa1^−/−^ piglets in the control group (Figure 3). Administration of ATRA to pregnant sows increased the AQP5 expression and decreased the SFTPC expression in the lungs of neonatal Hoxa1^−/−^ piglets compared with neonatal Hoxa1^−/−^ piglets in the control group. Neonatal Hoxa1^−/−^ piglets delivered by sows supplemented with ATRA at dpc 14 had higher AQP5 levels and lower SFTPC levels in the lungs than neonatal Hoxa1^−/−^ piglets born to sows administrated with ATRA at dpc 12 or 13, respectively.

### 3.4. Development of Microvessels in the Lungs

**Figure 4:**
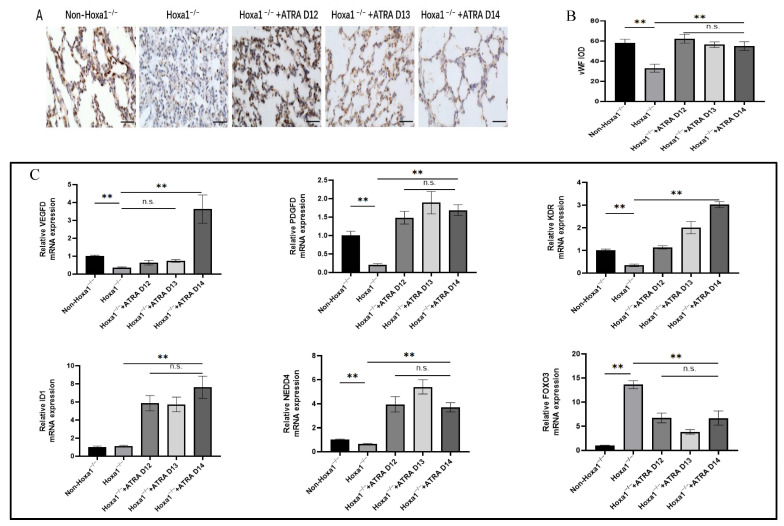
Results of immunohistochemical staining. (**A**) representative photomicrographs (magnification, 400×; scale bar, 15 μm). (**B**) mean microvessel density in the lung tissues. (**C**) microvascular-development-related genes. VEGFD: vascular endothelial growth factor D. PDGFD: platelet-derived growth factor-D. KDR: kinase insert domain receptor. ID1: inhibitor of differentiation 1. NEDD4: neuronal precursor cell-expressed developmentally downregulated 4. FoxO3: forkhead box O3. All data are expressed as mean ± SEM. ** *p* < 0.01, n.s., not significant.

In order to explore lung angiogenesis, immunohistochemical staining was performed using vWF as a specific marker for microvessel endothelial cells (Figure 4A). Hoxa1 mutation decreased the integrated optical density (IOD) of stained lung blood vessels, and neonatal Hoxa1^−/−^ piglets had lower (*p* < 0.01) IOD values than neonatal non-Hoxa1^−/−^ piglets in the control group (Figure 4B). Maternal administration with ATRA increased the IOD values of neonatal Hoxa1^−/−^ piglets in the experimental groups; neonatal Hoxa1^−/−^ piglets from the three experimental groups had higher (*p* < 0.01) IOD values than neonatal Hoxa1^−/−^ piglets from the control group, respectively, and no significant differences were found in the IOD values of neonatal Hoxa1^−/−^ piglets among three experimental groups.

The Hoxa1 mutation and maternal ATRA administration altered the relative expression of microvessel-development-related genes in the lungs (Figure 4C). The results indicate that in the control group, neonatal Hoxa1^−/−^ piglets had lower expressions of VEGFD (*p* < 0.01), PDGFD (*p* < 0.01), KDR (*p* < 0.01), and NEDD4 (*p* < 0.01), but higher expression of FOXO3 (*p* < 0.01) in the lungs compared with neonatal non-Hoxa1^−/−^ piglets. Maternal administration with ATRA elevated the levels of VEGFD, PDGFD, KDR, ID1, and NEDD4, but reduced the level of FOXO3 in the lungs. Neonatal Hoxa1^−/−^ piglets born to sows supplemented with ATRA at dpc 14 had higher (*p* < 0.01) levels of VEGFD, PDGFD, KDR, ID1, and NEDD4 and lower (*p* < 0.01) levels of FOXO3 in the lungs than neonatal Hoxa1^−/−^ piglets delivered by sows in the control group.

## 4. Discussion

The lungs are vital in keeping animals alive, and the abnormal development of the lungs may lead to poor health, low performance, or even death. The mutation of genes exerts adverse effects on lung health by impairing immune function, morphological structure, and blood vessel development in the lungs. The Pellino 1 (Peli1) mutation exhibits an impaired innate cytokine production, and Peli1^−/−^ mice have been shown to have lower levels of IFN-α and TNF-α than Peli1^+/+^ mice [27]. High levels of IFN-γ or (and) TNF-α play crucial roles in viral and bacterial clearance and infant health protection [28,29,30]. It is reported that the treatment of dendritic cells with ATRA promoted NK cell-derived IFN-γ production [31]. Furthermore, IL-8 has its special functions in T-lymphocyte chemotaxis [32] and neutrophil activation [33], and the level of IL-8 positively correlates to the concentration of TNF-α [34]. Therefore, certain levels of IFN-γ, TNF-α, and IL-8 are helpful in eradicating pathogens, and extremely low levels of these cytokines can affect the normal development of the lungs. The findings of this experiment indicate that the Hoxa1 mutation decreased the levels of IFN-γ, TNF-α, and IL-8 in the lungs. Administration with ATRA to Hoxa1^+/−^ pregnant sows at a level of 4 mg/kg body weight on dpc 14 increased the levels of IFN-γ, TNF-α, and IL-8 in the lungs of neonatal Hoxa1^−/−^ piglets when compared to neonatal Hoxa1^−/−^ piglets in the control group, and the elevated IFN-γ, TNF-α, and IL-8 alleviated the symptoms of dyspnea and abdominal respiration in neonatal Hoxa1^−/−^ piglets delivered by sows who had been administered ATRA at a dose of 4 mg/kg body weight on dpc 14.

The proper supply of nutrients plays vital roles during organogenesis, because the installation of blood vessel networks is mandatory during the embryonic period. ATRA can regulate lung development and regeneration through the regulation of pulmonary vasculogenesis and angiogenesis [35,36], owing to the fact that ATRA is a transcriptionally active agent [37,38], and maternal administration with ATRA was shown to stimulate alveologenesis in a model of nitrofen-induced pulmonary hypoplasia [39]. The results of this experiment demonstrate that maternal supplementation with ATRA increases the airspace area and the IOD, and decreases the thickness of the septal wall in the lungs of neonatal Hoxa1^−/−^ piglets compared to the lungs of neonatal Hoxa1^−/−^ piglets in the control group.

The increase in AT2 cells and the decrease in AT1 cells may lead to the death of newborn animals, owing to the imbalance between AQP5 and SFTPC expression [40]. AQP5 is selectively expressed in AT1 and plays an important role in water permeability [41]; the fetal lung can produce fluid to increase the amniotic fluid volume, and that is regulated by AQPs such as AQP5 [42,43]. SFTPC is produced predominantly by AT2 cells and is responsible for reducing pulmonary surface tension [44,45]. SFTPC can increase membrane permeability, and excessive expression of SFTPC causes the development of edemas by forcing large amounts of alveolar fluid into the lung tissue [46,47]. The expression of SFTPC can be altered by some cytokines, such as IFN-γ, because increasing IFN-γ could decrease SFTPC levels [48]. ATRA can induce AT2 proliferation [49,50], promote fetal AT2 differentiation to AT1, and enhance AQP5 expression [13]. The data in this study indicate that the Hoxa1 mutation disturbed the balance between AQP5 and SFTPC, and the administration of ATRA to pregnant sows improved the balance between AQP5 expression and SFTPC expression.

The normal development of pulmonary microvessels has an important impact on lung function. VEGFD, PDGFD, KDR, ID1, NEDD4, and FOXO3 are crucial genes in regulating the vascular network development of lung. The Hoxa1 mutation decreased (*p* < 0.01) the expression of VEGF, PDGFD, KDR, and NEDD4, but increased (*p* < 0.01) FOXO3 expression in the lungs of neonatal Hoxa1^−/−^ piglets, maternal administration with ATRA at 4 mg/kg body weight on dpc 14 elevated (*p* < 0.01) the expression of VEGF, PDGFD, KDR, and NEDD4, but decreased FOXO3 expression in the lungs of neonatal Hoxa1^−/−^ piglets compared to neonatal Hoxa1^−/−^ piglets in the control group. It is reported that VEGFD exerts important roles in the production and maturation of SFTPC, and SFTPC can prevents the collapse of alveolar cells. Down-regulating VEGFD expression may lead to shortness of breath and increase the rate of breathing [51]. Our study found that neonatal Hoxa1^−/−^ piglets from the control group had severe dyspnea owing to the Hoxa1 mutation and had lower VEGFD expression in the lungs than healthy neonatal non-Hoxa1^−/−^ piglets. This is similar to the report that infants with severe respiratory distress syndrome had significantly lower VEGFD expression in the lungs than healthy infants [52]. PDGFD can be expressed in many kinds of cells, including endothelial cells and vascular smooth muscle cells [53,54], and is closely related to angiogenesis and tissue fibrosis [55,56,57]. Downregulation of PDGFD will inhibit angiogenesis [58,59,60,61]. The Hoxa1 mutation also reduced PDGFD expression in the lungs of neonatal Hoxa1^−/−^ piglets with decreased micro-vessel density in the lungs. KDR participates in the branching morphogenesis processes, and the high expression level of KDR promoted vasculogenesis in the lungs [62]. Increasing the thickness of the alveolar septum can block angiogenesis by downregulating KDR expression, and the downregulation of KDR is prevented by treatment with RA [63]. Our study concluded with a similar result, that Hoxa1 mutation achieved a decreased KDR expression and thickened septal wall in the lungs of neonatal Hoxa1^−/−^ piglets, but maternal administration with ATRA elevated the KDR expression and reduced the thickness of the septal wall in the lungs of neonatal Hoxa1^−/−^ piglets. Downregulating NEDD4 can cause pulmonary edemas by increasing vascular permeability [64], and low NEDD4 expression can cause mice to die perinatally with a failure to breathe [65]. This is similar to our results, because pulmonary edema (Figure 1A) was also found in neonatal Hoxa1^−/−^ piglets with low NEDD4 expression, but maternal administration with ATRA reversed the congestion and swelling in the lungs of neonatal Hoxa1^−/−^ piglets (Figure 1B) and increased their survival rates. Previous studies have reported that the overexpression of FOXO3 results in microvascular endothelial apoptosis [66], and FOXO3 can significantly inhibit the migration of endothelial cells and the formation of tubes by suppressing the expression of angiogenesis genes such as PDGF. The decrease in FOXO3 expression enhanced the formation and maturation of blood vessels [67] and decreased the apoptosis of vascular endothelial cells [68]. The results of this experiment show that neonatal Hoxa1^−/−^ piglets had higher FOXO3 expression than the neonatal non-Hoxa1^−/−^ piglets in the control group, and the increase in FOXO3 expression inhibited the formation of blood vessels in the lung tissue of neonatal Hoxa1^−/−^ piglets by decreasing the density of the blood vessels. Maternal feeding with ATRA decreased the FOXO3 expression and increased the density of the microvessels in the lungs of neonatal Hoxa1^−/−^ piglets.

## 5. Conclusions

The Hoxa1 mutation causes the development of abnormal lung function by destroying the normal formation of alveoli and the pulmonary microvascular network of Hoxa1^−/−^ piglets. Maternal administration with ATRA at 4 mg/kg body weight on dpc 14 rescued the abnormal lung development of Hoxa1^−/−^ piglets by significantly increasing the IFN-γ concentration, the airspace area, the pulmonary microvessel density, and the expression of VEGFD, PDGFD, KDR, NEDD4, and AQP5, as well as by significantly decreasing the septal wall thickness and the expression of FOXO3 and SFTPC.

## Figures and Tables

**Table 1:** Treatment of pregnant sows in different groups.

	All-Trans Retinoic Acid Offered to Sows at Different DPC	Levels of All-Trans Retinoic Acid (mg/kg Body Weight)	Number of Sows in Different Groups
Control group	0	0	6
Experimental group 1	12	4	2
Experimental group 2	12	5	2
Experimental group 3	12	6	2
Experimental group 4	13	4	2
Experimental group 5	13	5	2
Experimental group 6	13	6	2
Experimental group 7	14	4	2
Experimental group 8	14	5	2
Experimental group 9	14	6	2

**Table 2:** Information on the primers.

Genes	Accession Numbers	Primer Sequences (5′~3′)	Product Size (bp)
VEGFD	XM_001928382.5	F AGATCCCAGAAGAAGATGGATGT	198
		R ACAGACACACTCGCAACGAT	
PDGFD	XM_021062718.1	F TCAGTAACGGACCCCACTCT	198
		R GCCGGTCCAGGTCAACTTT	
KDR	XM_013997943.2	F CTGCCTACCTCACCTGTTTC	100
		R ACTGACTTAGAGAGTACCTGAT	
ID1	NM_001244700.1	F GATCGCATCTTGTGTCGCTG	101
		R GGTGCTTGGAAGGACCAGAG	
NEDD4	XM_021094899.1	F TCTTGGGAGCTAGACTTTGAATCC	146
		R AAAGAGGAACATCCACTTGACCT	
FOXO3	XM_021084231.1	F CAGCAGCACAGTGTTTGGAC	120
		R AGTGTCTGGTTGCCGTAGTG	

**Table 3:** Level of inflammatory factors in the lungs of neonatal piglets from different treatment groups.

	IFN-γ (pg/mL)	TNF-α (pg/mL)	IL-8 (pg/mL)
Non-Hoxa1^−/−^	63.98 ± 7.54 ^a^	19.63 ± 1.24 ^c^	75.16 ± 1.13 ^bc^
Hoxa1^−/−^	33.24 ± 3.44 ^b^	18.73 ± 0.68 ^c^	39.18 ± 0.13 ^c^
Hoxa1^−/−^ + ATRA D12	38.36 ± 2.18 ^b^	22.85 ± 2.12 ^ab^	90.31 ± 0.13 ^b^
Hoxa1^−/−^ + ATRA D13	46.95 ± 3.74 ^b^	27.48 ± 1.14 ^a^	251.65 ± 22.40 ^a^
Hoxa1^−/−^ + ATRA D14	62.43 ± 4.52 ^a^	20.57 ± 1.50 ^bc^	73.49 ± 5.38 ^bc^

Note: non-Hoxa1^−/−^: neonatal non-Hoxa1^−/−^ piglets born by sows in the control group; Hoxa1^−/−^: neonatal Hoxa1^−/−^ piglets born to sows in the control group; Hoxa1^−/−^ + ATRA D12: neonatal Hoxa1^−/−^ piglets born to sows supplemented with ATRA at dpc 12, Hoxa1^−/−^ + ATRA D13: neonatal Hoxa1^−/−^ piglets born to sows supplemented with ATRA at dpc 13, Hoxa1^−/−^ + ATRA D14: neonatal Hoxa1^−/−^ piglets born to sows supplemented with ATRA at dpc 14. Means within a column followed by different lowercase letters differ significantly (*p* < 0.05).

## Data Availability

The original contributions presented in the study are included in the article, and further inquiries can be directed to the corresponding author.
